# Cell Aging of Mouse Gastrointestinal Tract Observed by Light and Electron Microscopic Radioautography

**DOI:** 10.14740/gr617e

**Published:** 2014-07-31

**Authors:** Tetsuji Nagata

**Affiliations:** Department of Anatomy and Cell Biology, Shinshu University School of Medicine, Matsumoto 390-8621, Japan. Email: nagatas@cnet.ne.jp

**Keywords:** Cell aging, Gastrointestinal tract, Electron microscopy, Radioautography, DNA, RNA, Protein, Lipid, Synthesis

## Abstract

The term “cell aging” initially means how the cells change due to their aging. There are two meanings, i.e. how a cell changes when it is isolated from original animals such as *in vitro* cells in cell culture, otherwise how all the cells of an animal change *in vivo* due to the aging of the individual animal. We have been studying the latter changes from the viewpoint of the cell nutrients, the precursors for the macromolecular synthesis such as deoxyribonucleic acid (DNA), ribonucleic acid (RNA), proteins, glucides and lipids, which are incorporated and synthesized into various cells of individual animals. Therefore, this article deals with only the cell aging of animal cells *in vivo*, how the metabolism, i.e. incorporations and syntheses of respective nutrient precursors in various kinds of cells change due to the aging of individual experimental animals such as mice by means of microscopic radioautography to localize the RI-labeled precursors. The incorporations and syntheses of various precursors for macromolecules such as DNA, RNA, proteins, glucides, lipids and others in various kinds of cells of various organs in the gastrointestinal tract such as the mouth, esophagus, stomach and intestines are reviewed referring many original papers already published from our laboratory during these 60 years since the late 20th century.

## Introduction

The term “cell aging” initially means how the cells change due to their aging. It contains two meanings; one is how a cell changes when it is isolated from *in vivo* original animals such as *in vitro* cells in cell culture, while the other means how all the cells of an animal change *in vivo* due to the aging of the individual animal. I had first studied the meaning of cell aging many years ago (more than 50 years) how a cell changed when it was isolated from original experimental animals such as mice and rats in cell culture [1-3], and then moved to the study on the latter cell aging, i.e. how all the cells of an experimental animal change *in vivo* due to the aging of the individual prenatal and postnatal animal [4-8].

Recently, we have been studying the aging changes from the viewpoint of the cell nutrients that were incorporated and synthesized into various cells in individual animals during their aging [9]. Therefore, this article deals with only the cell aging of animal cells *in vivo*, how the metabolism, i.e. incorporations and syntheses of respective nutrients, the macromolecular precursors, in various kinds of cells change due to the aging of individual experimental animals such as mice and rats by means of microscopic radioautography (RAG). Among the incorporations and syntheses of various nutrients such as deoxyribonucleic acid (DNA), ribonucleic acid (RNA), proteins, glucides, lipids and others in various kinds of cells of various organ in respective organ systems such as skeletal, muscular, circulatory, digestive, respiratory, urinary, reproductive, endocrine, nervous and sensory systems, this paper should review only the digestive system focusing on the gastrointestinal tract referring many original papers already published from our laboratory.

### RAG

In order to observe the localizations of the incorporations and syntheses of various nutrients synthesizing macromolecules in the animal bodies such as DNA, RNA, proteins, glucides and lipids in various kinds of cells of various organs in respective organ systems such as skeletal, muscular, circulatory, digestive, respiratory, urinary, reproductive, endocrine, nervous and sensory systems, we employed the specific techniques developed in our laboratory during these 50 years [10]. The technique employs various RI-labeled compounds and is designated as RAG.

To demonstrate the localizations of macromolecular synthesis by using such RI-labeled precursors as ^3^H-thymidine for DNA, ^3^H-uridine for RNA, ^3^H-leucine for protein, ^3^H-glucosamine or ^35^SO4 for glucides and ^3^H-glycerol for lipids are divided into macroscopic RAG and microscopic RAG. The techniques employ both the physical techniques using RI-labeled compounds and the histochemical techniques treating tissue sections by coating sections containing RI-labeled precursors with photographic emulsions and processing for exposure and development. Such techniques can demonstrate both the soluble compounds diffusible in the cells and tissues and the insoluble compounds bound to the macromolecules [11]. As a result, specimens prepared for electron microscopic radioautography (EM RAG) are very thick, as thick as 10 - 15 mµ, and should be observed with high-voltage electron microscopes in order to obtain better transmittance and resolution [12, 13]. Such radioautographic techniques in details should be referred to other literature [10]. On the other hand, the systematic results obtained by RAG should be designated as RAG, or science of RAG [14-16].

This article deals with the results dealing with the radioautographic changes of individual cell by aging that should be included in radioautographology.

### Macromolecular synthesis

The human body, as well as the bodies of any experimental animals such as mice and rats, consists of various macromolecules. They are classified into nucleic acids (both DNA and RNA), proteins, glucides and lipids, according to their chemical structures. These macromolecules can be demonstrated by specific histochemical staining for respective molecules such as Feulgen reaction [17] that stains all the DNA molecules contained in the cells. Each compound of macromolecules such as DNA, RNA, proteins, glucides lipids can be demonstrated by respective specific histochemical stainings [18] and such reactions can be quantified by microscpectrophotometry using specific wave-lengths demonstrating the total amount of respective compounds [19]. To the contrary, RAG can only demonstrate the newly synthesized macromolecules such as synthetic DNA or RNA or proteins depending upon the RI-labeled precursors incorporated specifically into these macromolecules such as ^3^H-thymidine into DNA or ^3^H-uridine into RNA or ^3^H-amino acid into proteins [10].

Concerning to the newly synthesized macromolecules, the results of recent studies in our laboratory by the present author and co-workers should be reviewed in this article according to the classification of macromolecules such as nucleic acids (DNA, RNA), proteins, glucides, lipids and others, as follows.

## The DNA Synthesis

The DNA contained in cells can be demonstrated either by morphological histochemical techniques staining tissue sections such as Feulgen reaction or by biochemical techniques homogenizing tissues and cells. To the contrary, the synthetic DNA or newly synthesized DNA but not all the DNA can be detected as macromolecular synthesis together with other macromolecules such as RNA or proteins in various organs of experimental animals by either morphological or biochemical procedures employing RI-labeled precursors. We have studied the sites of macromolecular synthesis in almost all the organs of mice during their aging from prenatal to postnatal development to senescence by means of microscopic RAG [20-28]. The results should be here described according to the order of organ systems in anatomy or histology.

### The DNA synthesis in the gastrointestinal tract of mouse

The digestive system consists of the gastrointestinal tract and the digestive glands. The gastrointestinal tract can be divided into several portions, the oral cavity, the pharynx, the esophagus, the stomach, the small and large intestines and the anus, while the digestive glands consist of both the large glands such as the salivary glands, the liver and the pancreas and the small glands affiliated to the digestive tracts in the gastrointestinal walls such as the gastric glands, intestinal glands of Lieberkuehn or duodenal glands of Brunner. We have published many papers from our laboratory dealing with the macromolecular synthesis of respective digestive organs from the oral cavity to the gastrointestinal tracts and the digestive glands [10, 20, 22, 29-41]. The outline of the results concerning to the synthetic DNA in the digestive organs should be here described in the order of systematic anatomy and special histology as follows.

#### The DNA synthesis in the oral cavity

**Figure 1:**
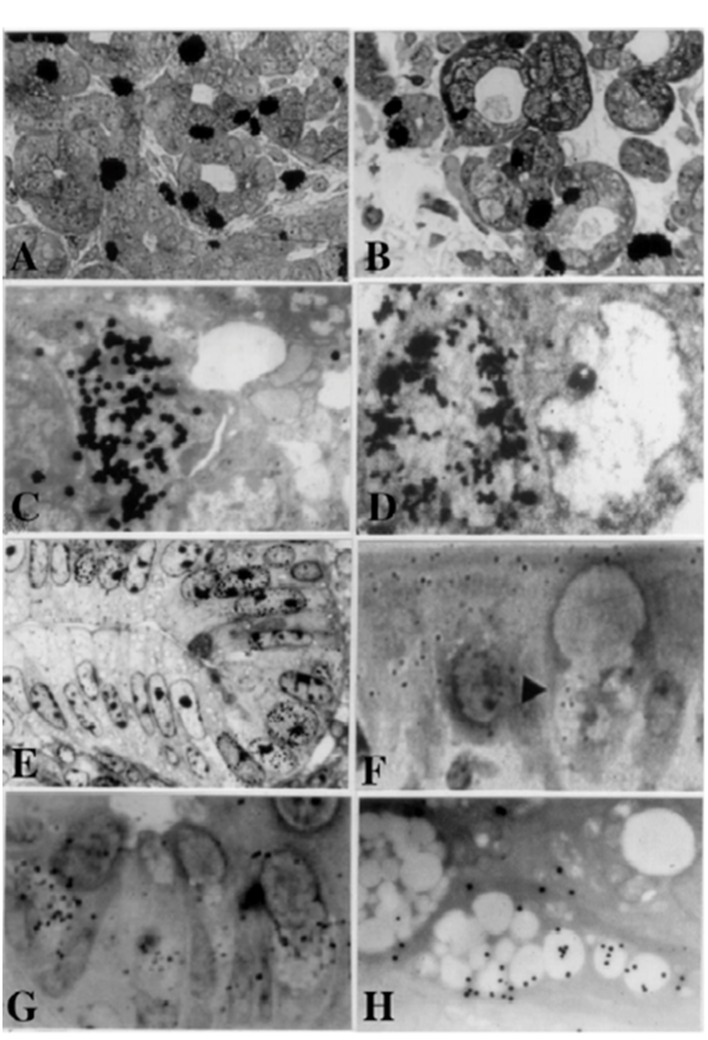
From Nagata, T.: Radioautographology, General and Special. In, Progr. Histochem. Cytochem. Vol. 37, No. 2, p. 118, 2002, Figure 5. LM and EM RAG of the digestive organs. Urban & Fischer, Jena, Germany. (A) LM RAG of the submandibular gland of male mouse embryonic day 19 labeled with ^3^H-thymidine consisted with the glandular acini and duct system (× 500). The duct system was composed of juxtaacinar cells (JA), intercalated duct cells (ICD) and striated duct cells (ICD). Many labeled developing acinar cells (AC), JA and ICD cells were observed. (B) LM RAG of the submandibular gland at postnatal day 3, labeled with ^3^H-thymidine (× 500). There were more JA cells and secretory granules than those of former stage (Fig. 1A). (C) EM RAG of an ICD cell of a mouse at postnatal day 3, labeled with ^3^H-thymidine observed by electron microscopy (× 10,000). Many silver grains are observed over the nucleus of an ICD. (D) EM RAG of the esophageal epithelial cells of a newborn mouse at postnatal day 1, labeled with ^3^H-thymidine (× 10,000). Many silver grains are observed over one of the nuclei at left. (E) LM RAG of the colonic epithelial cells of a mouse embryo at fetal day 19, labeled with ^3^H-thymidine (× 800). Many silver grains are observed over the nuclei of several epithelial cells in the bottom of the crypt. (F) LM RAG of the ileum epithelial cells labeled with ^3^H-glucosamine of an old mouse at postnatal 6 months (× 1,000). Many silver gains are localized over the Golgi region of the three goblet cells as well as over the cytoplasm of several absorptive columnar epithelial cells. (G) LM RAG of the colonic epithelial cells of a mouse at postnatal month 1, labeled with ^35^SO_4_ in vitro and radioautographed (× 1,000). (H) EM RAG of a goblet cell in the deeper crypt of the colonic epithelial cells of an adult mouse after injection of ^35^SO_4_ and radioautographed (× 4,800). Many silver grains are observed over the Golgi region and mucous droplets of the goblet cell, demonstrating the incorporation of radiosulfate into sulfomucins.

The oral cavity consists of the lip, tongue, tooth and the salivary gland. The DNA synthesis of mucosal epithelia of the two upper and lower lips and the tongues of aging mice from fetal day 19 to postnatal 2 years were studied by LM and EM RAG labeled with ^3^H-thymidine. However, the results were not analyzed statistically and would not be shown in this article. The salivary glands consist of the submandibular gland, the sublingual gland and the parotid gland that show the DNA syntheses in the acinar cells (Fig. 1A, 1B). As for the DNA syntheses in these cells, please refer to the original papers which were published already [21, 22, 31, 33, 34].

#### The DNA synthesis in the esophagus

The esophagus is the characteristic digestive tract including all the layers, the mucous membrane covered with the stratified squamous epithelia, the submucosa, the muscular layer and the serosa or adventitia. We studied the DNA synthesis of the esophagus of aging mice labeled with ^3^H-thymidine by LM and EM RAG [42, 43]. The labeled cells were mainly found in the basal layer of the esophageal epithelium (Fig. 1D). By electron microscopy, the nuclei and nucleoli of labeled cells were larger than those of unlabeled cells, but contained fewer cell organelles [43]. The labeling indices in respective aging groups showed a peak at postnatal day 1 and decreased with aging keeping a constant level around a few percentage from 6 months to 2 years after birth.

#### The DNA synthesis in the stomach

The stomach consists of the mucosa covered with the surface epithelia consisting of the columnar epithelia, including the gastric glands, the submucosa, the muscular layer and the serosa. As for the turnover of fundic glandular cells shown by ^3^H-thymidine RAG, it was extensively investigated with LM RAG by Leblond and co-workers [44-46]. They demonstrated that the DNA synthesis in the stomach increased at perinatal stages and decreased due to aging and senescence. However, the activity never reached zero but low activity continued until senescence. We studied the macromolecular synthesis including DNA, RNA, protein and glycoproteins in the gastric mucosa of both human and animal tissues by LM and EM RAG. As for the DNA synthesis, we obtained the same results as previously reported [46]. Therefore, the minute details will be here omitted.

#### The DNA synthesis in the intestines

The intestines of mammals are divided into two portions, small and large intestines, which can be further divided into several portions, the duodenum, the jejunum, the ileum, the cecum, the colon and the rectum. The intestinal tracts in any portions consist of the mucosa covered with columnar epithelial cells including absorptive and secretory cells, the submucosa, the smooth muscular layer and the serosa. We studied the macromolecular synthesis by LM and EM RAG mainly in the epithelial cells [10]. The DNA synthesis of small and large intestines of mice were studied by ^3^H-thymidine RAG (Fig. 1E). The cells labeled with ^3^H-thymidine were localized in the crypts of both small and large intestines, a region defined as the proliferative zone. In the colon of aging mice from fetal to postnatal 2 years, the labeled cells in the columnar epithelia were frequently found in the perinatal groups from embryo to postnatal day 1. However, the labeling indices became constant from the suckling period until senescence [47, 48].

**Figure 2:**
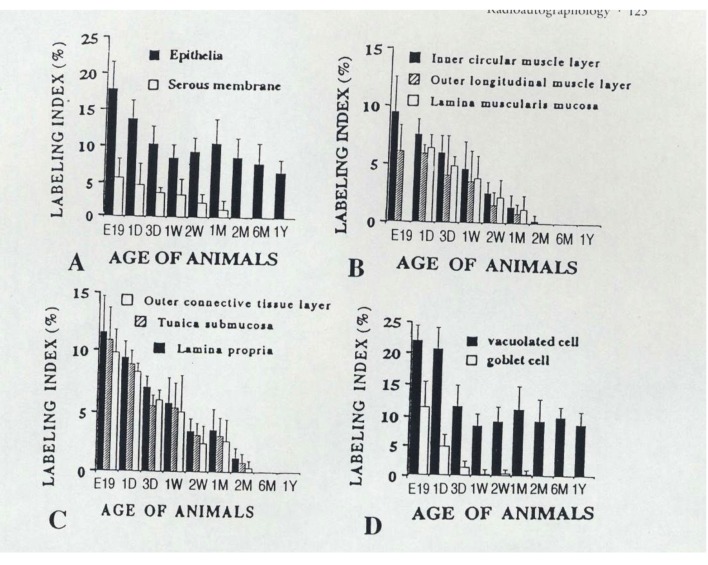
From Nagata, T.: Radioautographology, General and Special. In, Progr. Histochem. Cytochem. Vol. 37, No. 2, p. 123, 2002, Urban & Fischer, Jena, Germany. Histogram showing aging changes of average labeling indices in respective tissue layers and cells of mouse colons at various ages from embryo to postnatal year 1, labeled with ^3^H-thymidine.

On the other hand, we examined the labeling indices of respective cell types in each layer of mouse colon such as columnar epithelial cells, lamina propria, lamina muscularis mucose, tunica submucosa, inner circular muscle layer, outer longitudinal muscle layer, outer connective tissue and serous membrane of the colon, and found that most labeling indices decreased after birth to 2 months except the epithelial cells which kept constant value to senescence [49-51] (Fig. 2).

Similar results were also obtained from the cecal tissues of mouse by LM and EM RAG. We also studied immunostaining for PCNA/cyclin and compared to the results obtained from RAG [48]. We fixed the colonic tissues of litter mice of six aging groups from the embryonic day 19, to newborn postnatal day 1, 5 and 21, adult 2 months and senescent 12 months in methacarn and immunostained the colonic epithelium for cyclin proliferating nuclear antigen (PCNA/cyclin), which appeared from G1 to S phase of the cell cycle, with the monoclonal antibody and the avidin-biotin peroxidase complex technique. The immunostaining positive cells were localized in the crypts of colons similarly to the labeled cells with ^3^H-thymidine by RAG, a region defined as the proliferative zone. The positive cells in the columnar epithelia were frequently found in the perinatal groups from embryo to postnatal day 1, and became constant from postnatal day 5 until senescence. Comparing the results by immunostaining with the labeling index by RAG, it was found that the former was higher in each aging group than the latter. The reason for the difference should be due to that PCNA/cyclin positive cells included not only S-phase cells but also the late G1 cells.

## The RNA Synthesis

The RNA contained in cells can be demonstrated either by morphological histochemical techniques staining tissue sections such as methyl green-pyronin staining or by biochemical techniques homogenizing tissues and cells. To the contrary, the synthetic RNA or newly synthesized RNA but not all the RNA in the cells can be detected as macromolecular synthesis together with other macromolecules, such as DNA or proteins in various organs of experimental animals by either morphological or biochemical procedures employing RI-labeled precursors. We have studied the sites of macromolecular synthesis in almost all the organs of mice during their aging from prenatal to postnatal development to senescence by means of microscopic RAG, one of the morphological methods [20-22, 24-26, 28]. The results obtained from RNA synthesis should be here described according to the order of organ systems in anatomy or histology. In contrast to the results obtained from DNA synthesis of almost all the organs, we have studied only several parts of the organ systems. The skeletal system, the muscular system or the circulatory system were not so much studied.

### The RNA synthesis in the digestive system

We have mainly studied the digestive system, but not all the digestive organs yet are concerning to the RNA synthesis. Study on RNA synthesis was carried out only on the small intestines.

#### The RNA synthesis in the intestines

We studied the RNA synthesis of the small intestines of mice after feeding or refeeding under the restricted conditions [52]. Five groups of ddY mice, each consisting of five individuals, totaling 25, were injected with ^3^H-uridine, an RNA precursor, and killed at different time intervals after feeding. The animals of the first group were injected with ^3^H-uridine at 9 am and fed at 10 am for 30 min and killed at 11 am 1 h after the feeding and 2 h after the injection, the second group was killed at 1 pm 3 h after feeding and 4 h after the injection, the third group at 5 pm, 7 and 8 h later, the fourth group at 9 am on the next day 23 and 24 h later, and finally the fifth group at 1 pm on the next day 3 h after refeeding and 28 h after the injection. Then, the jejunums obtained from each animal were prepared for isolated cell radioautograms according to previous report [53]. The results demonstrated that the grain counts in mononucleate villus cells reached the maximum (20 - 30 grains per cell) 4 h after injection and decreased (10 - 20/cell) after 28 h, while the counts in mononucleate villus cells only increased gradually from 4 h (10/cell) to 28 h (20/cell). In contrast to this, the grain counts of binucleate cells which appeared in villus cells increased parallel to the mononucleate villus cells (10 - 20/cell). It was concluded that the RNA synthesis in the jejunal epithelial cells was high in the following order: mononucleate crypt cells, binucleate cells and mononucleate villus cells. These results revealed that the feeding or refeeding affected the RNA synthesis of the intestinal epithelial cells [53].

We also studied intracellular localization of mRNA in adult rat hepatocytes localizing over the peroxisomes by means of *in situ* hybridization technique [54-56]. However, its relationship to the aging of animals has not yet been studied.

## The Protein Synthesis in the Gastrointestinal Tract

We have studied the protein synthesis of the stomach and the intestines in the digestive tracts of mice and rats.

### Protein synthesis in the stomach

We formerly observed the secretion process in G cells by EM RAG using ^3^H-amino acid [57-59]. When the stomach tissues were taken out from the adult Wistar rats at postnatal month 1 and were labeled with either ^3^H-glutamic acid or ^3^H-glycine *in vitro* at varying time intervals, silver grains in the EM radioautograms appeared first over the Golgi zones, then migrated to secretory granules and were stored in the cytoplasm, suggesting the secretory kinetics. We also studied the mechanism of serum albumin passing through the gastric epithelial cells into the gastric cells by EM RAG [59]. When adult Wistar rat stomach tissues were labeled with ^132^I-albumin *in vitro* at varying time intervals, silver grains in the radioautograms appeared over rough endoplasmic reticulum within 3 min, then moved to the Golgi apparatus in 10 min, and on to secretory granules and into the lumen in 30 min, suggesting the pathway of serum albumin absorption from the blood vessels through the gastric mucous epithelial cells into the gastric lumen [59]. These results demonstrated that the stomach cells of adult rats synthesized proteins and secreted. However, aging changes of these proteins synthesis between the young and senescent animals were not yet completed.

### Protein synthesis in the intestines

We first studied the incorporations of ^3^H-leucine and ^3^H-tryptophane in mouse small intestines in connection to the binuclearity before and after feeding [60, 61]. The results showed that the incorporations of both amino acids were greater in binucleate intestinal epithelial columnar cells than mononucleate villus and crypt cells at both before and after feeding. However, the aging changes of these incorporations were not yet studied.

## The Glucide Synthesis

The glucides found in animal cells and tissues are composed of various low-molecular sugars such as glucose or fructose called monosaccharides which form compounds of polysaccharides or complex mucopolysaccharides connecting to sulfated compounds. The former are called simple polysaccharides, while the latter mucopolysubstances. Thus, the glucides are chemically classified into three groups, monosaccharides such as glucose or fructose, disaccharides such as sucrose and polysaccharides such as mucosubstances. However, in most animal cells polysaccharides are much more found than monosaccharides or disaccharides. The polysaccharides can be classified into two, i.e. simple polyscaccharides and mucosubstances. Anyway, they are composed of various low-molecular sugars that can be demonstrated by either histochemical reactions or biochemical techniques. To the contrary, the newly synthesized glucides but not all the glucides in the cells and tissues, can be detected as macromolecular synthesis together with other macromolecules such as DNA, RNA or proteins in various organs of experimental animals by either morphological or biochemical procedures employing RI-labeled precursors. We have studied the sites of macromolecular synthesis in almost all the organs of mice during their aging from prenatal to postnatal development to senescence by means of microscopic RAG [10, 20-22, 31, 62, 63]. The results obtained from glucides synthesis are described according to the order of organ systems in anatomy or histology. In contrast to the results obtained from DNA synthesis of almost all the organs, we have studied only several parts of the organ systems.

### The glucide synthesis in the digestive system

Among several digestive organs in the gastrointestianl tracts and the digestive glands, we have studied the glucide synthesis of the stomach and the intestines in the gastrointestinal tracts as well as the salivary gland, the liver and the pancreas in the digestive glands of aging mice.

#### The glucide synthesis in the oral cavity

In the oral cavity, we studied the incorporations of ^3^H-glucosamine in the submandibular glands of ten groups of litter mice at various ages. The animals from embryonic day 19, postnatal day 1, 3, 7, 14, and 1, 3, 6 months to 1 and 2 years were killed after administration of ^3^H-glucosamine and the submandibular glands were processed for LM and EM RAG [10, 64]. The results showed that the silver grains appeared over the endoplasmic reticulum, Golgi apparatus and the secretory granules of the acinar cells, demonstrating the glycoprotein synthesis in these cells. Grain counting revealed that the counts increased from the fetal stage at embryonic day 19 to postnatal day 1 to 3, 7, 14, reaching the peak at day 14, then decreased to month 1, 3, 6, to year 1 and 2, showing the aging changes, inverse proportion to DNA synthesis of these cells.

The sulfate uptake and accumulation in sulfomucin in several digestive organs of mice were also studied by light microscopic RAG [65-67]. Two litters of normal ddY mice 30 days after birth, each consisting of three animals, were studied. One litter of animals was killed at 30 min after the intraperitoneal injections with phosphate buffered Na_2_^35^SO_4_, and the other litter of animals were killed at 12 h after the injections. Then the submandibular glands and the sublingual glands were taken out, fixed, embedded in epoxy resin, sectioned, radioautographed and analyzed by light microscopy. As a result, many silver grains were observed on serous cells of the salivary glands at 30 min and 12 h after the injections (10 - 20/cell). The numbers of silver grains at 30 min were less than those at 12 h. From the results, it was concluded that glycoprotein synthesis was demonstrated in both the submandibular and sublingual glands by radiosulfate incorporation. In the salivary glands the silver grains were more observed in serous cells than mucous cells at 30 min, while in mucous cells more at 12 h than 30 min after the injection. These results show the time difference of glycoprotein synthesis in the two salivary glands, showing inverse proportion to DNA synthesis of these cells [10, 64].

#### The glucide synthesis of the stomach

When incorporation of radiosulfate into sulfated complex carbohydrate in rat stomach was studied by labeling with ^35^SO_4_ *in vivo*, silver grains appeared over the glandular cells of the pyloric gland but not those of the fundic gland, demonstrating the mucous synthesis in the former glands [65, 67]. The radiosulfate uptake and accumulation in the stomach of mouse were also studied by light microscopic RAG [67]. Two litters of normal ddY mice 30 days after birth, each consisting of three animals, were studied. One litter of animals were killed at 30 min after the intraperitoneal injections with phosphate buffered Na_2_^35^SO_4_ and the other litter animals were killed 12 h after the injections. Then the antrum and the fundus tissues of the stomachs were taken out. The tissues were fixed, dehydrated, embedded in epoxy resin, sectioned, radioautographed and analyzed. As a result, many silver grains were observed over the mucosa and submucosa of the stomach at 30 min after the injection. Then at 12 h after the injection silver grains were observed on some of the fundic glands. The numbers of silver grains observed in the stomach especially over the pyloric glands at 30 min (a few per cell) were less than those (several per cell) at 12 h. The results showed the time difference of glycoprotein synthesis in the stomach, showing inverse proportion to DNA synthesis [10, 65].

#### The glucide synthesis in the intestines

We also studied the aging changes of glucide synthesis by ^3^H-glucosamine uptake in the small intestines of mouse [47], and found that the silver grains in the ileum columnar epithelial cells were mainly localized over the brush borders and the Golgi regions in these cells (Fig. 1F). The grain counting revealed that the numbers of silver grains over the brush borders and cytoplasm of the columnar epithelial cells increased in the villi (10 - 15/cell) than in the crypts (1 - 2/cell) from 6 months up to 2 years due to aging. The grain counting in other cell types also revealed that the number of silver grains in goblet cells, basal granulate cells and Paneth cells increased by aging, but did not in the undifferentiated cells.

The glycoprotein synthesis in goblet cells, as well as in absorptive epithelial cells, was also studied using Na_2_^35^SO_4_ incorporation in the duodenums, the jejunums and the colons of adult mice at varying time intervals at 30, 60, and 180 min after the administration [65-67]. Silver grains were localized over the columnar absorptive cells and the goblet cells, especially over the Golgi regions and mucous granules of the goblet cells. By EM RAG the intracellular localization of silver grains in goblet cells was clearly shown in the Golgi apparatus. The results from grain counting revealed that the average grain counts were different in the upper and deeper regions of the crypts in the four portions and it was shown that silver grains over goblet cells in the lower region of the crypt transferred rapidly from 30 min to 180 min, while they transferred slowly in goblet cells in the upper region of the colonic crypt, leading to the conclusion that the rates of transport and secretion of mucous products of the goblet cells at these two levels in the crypts were different. By EM RAG silver grains first appeared over the Golgi zone at 30 min and then moved to the secretory granules at 60 and 180 min. The incorporation of Na_2_^35^SO_4_ into sulfated complex carbohydrate was investigated in the mouse small and large intestines by LM and EM RAG as well as in the submandibular glands and the stomachs. Quantitative differences have been observed in the relative uptake of radiosulfate in the various labeled cells of each organ. Incorporation by the colon in goblet cells exceeded that elsewhere in the deep goblet cells of the colonic crypts migration of label progressed during the time tested from the supranuclear Golgi region to the deep position of the goblet and then extended throughout the mucosubstance in the goblet in the superficial goblet cells of the colon. The radioautographic and cytochemical staining differences between secretory cells in the deeper region compared with the upper region of the colonic crypts are considered to reflect differences in the rate of transport of secretory products in the theca and the rate of secretion at the low levels in the crypt (Fig. 1G, 1H). These results showed the time differences of glycoprotein synthesis in respective organs. The sulfate uptake and accumulation in several mouse digestive organs were also studied by LM RAG. Two litters of normal ddY mice 30 days after birth, each consisting of three animals, were studied. One litter of animals was killed 30 min after the intraperitoneal injections with phosphate buffered Na_2_^35^SO_4_, and the other litter animals were killed 12 h after the injections. Then several digestive organs, the parotid gland, the submandibular gland, the sublingual gland, antrum and fundus of the stomach, the duodenum, the jejunum, the ileum, the cecum, the ascending colon and the descending colon were taken out and radioautographed. As the results, many silver grains were observed on villous cells and crypt cells of the small intestines and whole mucosa of the large intestines at 30 min after the injection. Then at 12 h after the injection silver grains were observed on mucigen granules of goblet cells in the small intestines and the large intestines. The numbers of silver grains observed in respective organs at 30 min were less than those at 12 h. From the results, it was concluded that the time difference of the glycoprotein synthesis was demonstrated in several digestive organs by radiosulfate incorporation, in reverse proportion to DNA synthesis. The total S contents in colonic goblet cells in upper and deeper regions of colonic crypts in aging mice were also analyzed by X-ray microanalysis [68, 69]. The results accorded well with the results from RAG [39] showing increase and decrease of mucosubstances in these cells due to development and aging to senescence.

## The Lipid Synthesis

The lipids found in animal cells are chemically composed of various low-molecular fatty acids. They are esters of high fatty acids and glycerol that can biochemically be classified into simple lipids and compound lipids such as phospholipids, glycolipids or proteolipids. The simple lipids are composed of only fatty acids and glycerol, while the latter are composed of lipids and other components such as phosphates, glucides or proteins. In order to demonstrate intracellular localization of total lipids, we can employ either histochemical reactions or biochemical techniques. To the contrary, the newly synthesized lipids but not all of the lipids in the cells can be detected as macromolecular synthesis similarly to the other macromolecules such as DNA, RNA, proteins or glucides in various organs of experimental animals by either morphological or biochemical procedures employing RI-labeled precursors. We have studied the sites of macromolecular synthesis in almost all the organs of mice during their aging from prenatal to postnatal development to senescence by means of microscopic RAG [10, 20-23, 31, 32, 62, 63, 70]. However, we have not studied the lipids synthesis so much as compared to other compounds. We have studied only a few organs of the digestive system.

### The lipids synthesis in the digestive system

We did not study the intestines but studied only the livers and the pancreases of aging mice at various ages demonstrating soluble compounds by means of cryo-fixation and dry-mounting RAG [23, 31, 71-73].

## The Intracellular Localization of the Other Substances

The substances other than macromolecules that can also be demonstrated by RAG are target tracers, not the precursors for the macromolecular synthesis. They are hormones such as ^3^H-methyl prednisolone [74], neurotransmitters and inhibitors such as ^14^C-bupranolol, a beta-blocking agent [75] or ^3^H-befunolol [76, 77], vitamins, drugs such as synthetic anti-allergic agent ^3^H-tranilast [78-81], hypolipidemic agent bezafibrate [82-85], calmodulin antagonist [86, 87] or anti-hypertensive agent ^3^H-benidipine hydrochloride [88], toxins, inorganic substances such as mercury [89] and others such as laser beam irradiation [11, 90]. The details are referred to the previous publication on the radioautographology [10, 91]. However, their relationships to the cell aging were not yet studied.

## Conclusion

From the results obtained, it was concluded that almost all the cells in various organs of all the organ systems of experimental animals at various ages from prenatal to postnatal development and senescence during the aging of cells and individual animals demonstrated to incorporate various macromolecular precursors such as ^3^H-thymidine, ^3^H-uridine, ^3^H-leucine, ^3^H-glucose or glucosamine, ^3^H-glycerol and others localizing in the nuclei, cytoplasmic cell organelles showing silver grains due to DNA, RNA, proteins, glucides, lipids and others those which the cells synthesized during the cell aging. Quantitative analysis carried out on the numbers of silver grains in respective cell organelles demonstrated quantitative changes, increases and decreases, of these macromolecular syntheses in connection to cell aging of respective organs. In general, DNA synthesis with ^3^H-thymidine incorporations in most organs showed maxima at perinatal stages and gradually decreased due to aging. To the contrary, the other synthesis such as RNA, proteins, glucides and lipids increased due to aging and did not remarkably decrease until senescence. Anyway, these results indicated that macromolecular synthetic activities of respective compounds in various cells were affected from the aging of the individual animals.

Thus, the results obtained from the various cells of various organs should form a part of special radioautographology that I had formerly proposed [10, 15], i.e. application of RAG to the aging of cells, as well as a part of special cytochemistry [12, 13], as was formerly reviewed. We expect that such special radioautographology and special cytochemistry should be further developed in all the organs in the future.
